# Current Challenges in Translational and Clinical fMRI and Future Directions

**DOI:** 10.3389/fpsyt.2019.00924

**Published:** 2020-01-08

**Authors:** Karsten Specht

**Affiliations:** ^1^Department of Biological and Medical Psychology, University of Bergen, Bergen, Norway; ^2^Mohn Medical Imaging and Visualization Centre, Haukeland University Hospital, Bergen, Norway; ^3^Department of Education, UiT/The Arctic University of Norway, Tromsø, Norway

**Keywords:** fMRI—functional magnetic resonance imaging, BOLD (blood oxygenation level dependent) signal, reliability, clinical fMRI, psychiatry

## Abstract

Translational neuroscience is an important field that brings together clinical praxis with neuroscience methods. In this review article, the focus will be on functional neuroimaging (fMRI) and its applicability in clinical fMRI studies. In the light of the “replication crisis,” three aspects will be critically discussed: First, the fMRI signal itself, second, current fMRI praxis, and, third, the next generation of analysis strategies. Current attempts such as resting-state fMRI, meta-analyses, and machine learning will be discussed with their advantages and potential pitfalls and disadvantages. One major concern is that the fMRI signal shows substantial within- and between-subject variability, which affects the reliability of both task-related, but in particularly resting-state fMRI studies. Furthermore, the lack of standardized acquisition and analysis methods hinders the further development of clinical relevant approaches. However, meta-analyses and machine-learning approaches may help to overcome current shortcomings in the methods by identifying new, and yet hidden relationships, and may help to build new models on disorder mechanisms. Furthermore, better control of parameters that may have an influence on the fMRI signal and that can easily be controlled for, like blood pressure, heart rate, diet, time of day, might improve reliability substantially.

## Introduction

Translational neuroscience is an important branch within the broad field of neuroscience. In the context of this opinion article, translational neuroscience will be seen as the attempt of bridging neuroscience, neuroimaging, and clinics for improving our understanding of symptoms and disorders, and for better diagnostics and treatments (1, 2).

This is not a new attempt. Neuroimaging, and in particular functional magnetic resonance imaging (fMRI), has been considered as a revolutionary tool for exploring the healthy and the diseased brain for more than two decades (3, 4). Consequently, since fMRI entered the scene in the early 1990s, it had seen an enthusiastic phase over the first two decades. However, after this period, neuroimaging—like almost any other psychological and medical sciences—was overrun by the replication crisis. Recent studies have estimated the reproducibility of psychological studies to be 39% or less and indicated a severe limitation of neuroimaging (fMRI) study reliability (5–9). Furthermore, the neurophysiological mechanisms behind the BOLD/fMRI signal are only partly understood, which makes it difficult to generalise results or to use it on an individual level for diagnostic purposes. Thereby impeding the impact of highly needed neuroscience studies on theoretical and methodological progress, and, last but not least, the clinical application of fMRI.

In the following, this article will critically discuss current strategies and developments within the field of neuroimaging and tries to indicate possible future directions.

### The Replication Crisis and Its Consequences

The neuroimaging research community has taken the “replication crisis” very seriously, like through the ReproNim initiative (10), and the Organisation of Human Brain Mapping (OHBM) announced in 2016 a new replication award, and put reproducibility high up on their agenda with several new best practice and data sharing initiatives (see, e.g., http://www.ohbmbrainmappingblog.com).

Jointly, psychology and neuroimaging suffer substantially from a lack of statistical power, meaning that the sample sizes are typically too small, and effect sizes are too low (11). This has not only been perceived as a critical challenge among scientists but has recently also received public attention. On the other hand, clinical applications require reliable single-case examinations but not group studies that may reliably show the general population effect but may vanish the information on interindividual and intraindividual variability. Consequently, the lack of information about the “naturally” occurring variability hinders the successful development of translational and clinical applications. Already in 2006, Paul Matthews and coworkers critically discussed the applicability of clinical fMRI for other applications than neurosurgical mapping (12). Although they wrote down their opinion more than a decade ago, it appears like that the development of clinical fMRI is in a “resting state,” as recently pointed out by O’Connor and Zeffiro (13). Presurgical mapping is still the only reliable and widely used clinical application of fMRI. The critical question is, why haven’t we yet achieved a breakthrough in clinical fMRI?

## Current Status

This article will critically discuss three aspects that are relevant to consider in the context of clinical fMRI: First, the fMRI signal itself, second, current fMRI praxis, and, third, the next generation of analysis strategies.

### The Bold Signal Perturbation

One of the major knowledge gaps in the field is the assumption that the fMRI signal, i.e., the underlying BOLD effect (BOLD = blood oxygenation level dependent), is *sufficiently* reliable and stable, where “sufficiently” has never been defined yet. It is of crucial importance to keep in mind that the BOLD signal represents only an indirect measure of neuronal activity, through a cascade of physiological processes, called neurovascular coupling. Consequently, the observed variability of the BOLD signal does not necessarily justify the conclusion that the underlying neuronal activity shows variability to the same degree. Scientifically speaking, the BOLD signal is a physiological response that only indirectly reflects neuronal activity, and which is easily and directly influenced by blood pressure, blood oxygenation, or any other parameters that have an effect on the vascular system, which in turn affect the balloon effect that generates the BOLD signal (14, 15). The corresponding balloon model became the most influential and mostly used model in fMRI research (15–17). It is a neurophysiological model that describes the neuronal and vascular mechanisms that cause the BOLD signal given a neuronal activity. It rests on the assumption that the BOLD signal is caused by changes in the blood volume, blood flow, and the oxygen extraction rate. It is widely accepted that these are the main parameters that determine the strength of the BOLD signal. The balloon model and its corresponding hemodynamic response function is, for example, an integral part of several analysis models of fMRI data, but also for measures of functional and effective connectivity, like dynamic causal modelling (DCM) (18).

However, it is less studied, how susceptible the BOLD signal is to endogenous and exogenous influences and individual variability of the underlying mechanisms. Hence, it might occur that a change in the BOLD signal is detected while the *true* neuronal activity and connectivity remains unchanged. It is known that hormones (like cortisol), blood pressures, body mass index, time of the day (circadian rhythm), time of the year, sleep duration, and age influence blood volume, blood flow, and other vascular parameter, and hence the BOLD signal (19–23). Whether the individual variability of these parameters has a significant influence on the BOLD signal is largely unknown. To give another example, using magnetic resonance spectroscopy (MRS), it has been shown that the individually varying concentration of the inhibitory neurotransmitter GABA is reflected in the amplitude and shape of the BOLD signal (24). Complementary, comparable effects have been shown for the excitatory neurotransmitter glutamate (25, 26). The list of those endogenous parameters can be continued, including parameter that may predominantly affect neuronal signal transmission or vascular processes. In other words, the BOLD signal is most likely not stable within and not necessarily comparable between subjects. These factors are just additional sources of variability of the fMRI signal that comes in addition to all other sources of noise that are affecting the measurement, like other environmental factors, thermal noise, noise of the measurement system itself, movements of the subjects, daylight length, temperature, and whether, to name a few, that may affect brain functions but also the stability of the MR system (23, 27, 28).

### Current fMRI Methods

#### Current Clinical Applications of fMRI

As outlined above, the only routinely used clinical application of fMRI is the presurgical mapping (see **Table 1**). This is mostly done in patients with brain tumours or epilepsy, since these diseases may cause substantial displacement of brain functions, and functional mapping with fMRI may help surgeons to localize important areas despite their unusual neuroanatomical localization (29). However, most of those clinical applications are task-related fMRI with simple paradigms, and the first reports date far back to the beginning of fMRI (30, 31).

**Table 1:** Schematic overview on reliability and current clinical applications of the different fMRI techniques.

	Reliability	Current clinical applications
**Task-related fMRI:** Localisation of functions	**Single subjects:** Reasonable good	Presurgical mapping; Occasionally single case studies
	Typical paradigms: Motor, Language, Memory	
	**Group studies:** Good; Various tasks are used	Psychiatry, Neurology
**Task-related fMRI:** Strength of activation	**Single subjects:** Poor - Limited -Depends on task, instructions & attention	Limited applicability
	**Group studies:** Limited - Good; Various Tasks	Psychiatry, Neurology
**Resting-state fMRI**	**Single subjects:** Poor – Limited - Good	Limited applicability
	Reliability depends mainly on analysis methods, i.e. different measures show different reliability, but it depends also on scan length, instruction, etc.	
	**Group studies:** Limited – Good	Psychiatry, Neurology
	Reliability depends mainly on analysis methods, i.e. different measures show different reliability, but it depends also on scan length, instruction, etc.	

One of the most common application is the localization of language areas and their lateralization, which is an essential information in the treatment of patients with epilepsy (32–38). In most occasions, this clinical application of fMRI shows comparable results as the invasive WADA test but might deviate in cases with atypical language dominance (39).

Good experience also exists for paradigms probing the localization of motor, sensory, and memory functions, which are often not only used for localization but also for predicting outcome (40–44). Nowadays, clinical fMRI is often combined with diffusion tensor imaging for localizing relevant fibre tracts (42).

In contrast to the presurgical mapping where it is sufficient enough to localize a function, any application in psychiatry, for instance, needs to focus on the strength of activations. Accordingly, there exists no routinely used clinical application of fMRI outside of the field of presurgical mapping due to the lack of sufficient reliability in the measurement of *individual* activation strength—for example task, instruction, and different levels of attention may influence reliability (45, 46). *Group studies*, by contrast, show a much higher reliability in detecting deviations in activation strength (47). Therefore, almost exclusively all fMRI studies in psychiatry have explored cohorts of patients (see **Table 1**). However, one possible way to circumvent this lack of reliability on the individual level has been recently suggested by Paek et al. in connection with a study on dementia by proposing repeated (baseline) measurements of the patients (48).

Another problem in clinical fMRI in psychiatry is the heterogeneity of patient populations. The disorders are often spectrum disorders with a continuum that ranges from normality to pathological (49), but also that varies between various symptoms and diagnosis, like between schizoaffective disorder, schizophrenia, bipolar disorder (50). Furthermore, the disorders often manifest in varies subtypes, and different studies may use different diagnostic criteria. Consequently, imaging results often differ substantially even on the level of group studies (51).

#### Is Resting-State fMRI the Solution?

While the first two decades of fMRI were mostly dominated by task-related fMRI, i.e., fMRI acquisitions while research subjects performed an active task, like a working memory, attention, or language task, the more recent years have seen an alternative approach, which is called “resting-state” fMRI (rs-fMRI). Here, research subjects are just scanned over a certain period without any concrete, active task—they are presumably “at rest.” Surprisingly, the measured BOLD signal that is measured during such an rs-fMRI examination is not random but fluctuates in a spatially and temporally systematic manner (52, 53). It has been shown that even in the absence of a concrete task, certain brain areas are forming networks through characteristic correlated fluctuations of the BOLD signal, called *resting-state* or *intrinsic networks*. These network patterns can be detected by focusing on low frequent (<0.01Hz) fluctuations of the BOLD signal, since these fluctuations propagate through the underlying neuronal network structures, indicating an information exchange within the networks even in the absence of a concrete task. It has been further shown that these networks are very similar across individuals (52, 53). They are therefore assumed to reflect some fundamental —trait- or biomarker-like —brain processes.

From resting-state data, it is possible to identify neuronal networks that show in their spatial organization a striking similarity with those networks that have been identified through task-related fMRI (54). These networks are often identifiable also on single subject levels, but depends on the method that is used for extracting the information (46). Since this discovery, there has been a tremendously interest in resting-state fMRI and the examination of the related intrinsic-brain networks and their dynamics (17, 55–62). One of the most investigated networks in this respect is the “default mode network” (DMN) (61, 63). The DMN network is related to processes, like mind wandering, intrinsically focused attention, daydreaming, etc. Interestingly, there is a counterpart to the DMN, which has been described under different names in the literature. Here, it will be called the “extrinsic mode network” (EMN) and represents a network for extrinsically focused attention (61).

One reason why rs-fMRI became such a popular tool in the field of neuroimaging is that it may allow studying cognitive functions even in the absence of a task, which would be an intriguing possibility for doing clinical fMRI, especially in cases where patients are severely affected like after a stroke or traumatic brain injury. Clinical applications of rs-fMRI are based on the assumption of certain interindividual and intraindividual stability of resting-state networks in healthy individuals to draw conclusions from observed deviations in patients. In order to increase comparability and to limit variability in data acquisition between studies, first sets of guidelines for standardized protocols have been developed (e.g., like the “Alzheimer’s disease neuroimaging initiative” (ADNI); http://adni.loni.usc.edu), and other initiatives are following their example and have started similar undertakings.

However, one major disadvantage of rs-fMRI is that rs-fMRI studies still vary in their acquisition methods and whether they are conducted on a 1.5T, 3T, or 7T MR. The typical approach in rs-fMRI is to do an fMRI scan of several minutes duration with a repetition time (TR) of typically 1–3 s. But between studies and labs, there are already at least three different types of instructions, asking the research subjects to either close their eyes, keep them open, or to fixate on a fixation cross. Although the differences between these three possible instructions are moderate, they are still measurable (64). Interestingly, the most reliable results for most but not all examined networks were achieved when subjects fixated on a fixation cross. It is, however, difficult to control how well an individual followed that instruction as eye-tracking devices or eye cameras are typically not installed inside of an MR scanner and especially not in clinical MR scanners. Furthermore, different TR times may also cause varying results, since periodic signals like heart rate variability or respiration rate might affect results differently (65, 66). Another factor that varies between different studies and also influences the results is the duration of the resting-state examination that roughly varies between a few minutes and up to 12 min and more. The reliability of specific rs-fMRI seems to improve with scan durations, and acceptable good reliability for both intrasession and intersession rs-fMRI might be around 12 min (67).

Furthermore, there are also still no standards of how rs-fMRI data should be analysed. Previous studies have applied a wide spectrum of rs-fMRI analysis strategies, with varying levels of reliability (46, 68). But progress has been made in standardizing some of the procedures for achieving across-site comparability (69). It is, however, beyond the scope of this article to review all the different methods, but, just as an example, it has been shown that different methods do have different reliability, like measures of the static functional connectivity networks against the temporal dynamics of these networks (70).

In addition, rs-fMRI studies are based on the assumption of the inherent stability of the underlying resting-state networks across time and individuals. In other words, one assumes a low intrasubject and intersubject variability with high sensitivity to clinical deviations. This assumption has, however, never been thoroughly tested and might not be justifiable. There are only sparse and inconsistent reports that resting-states are indeed resting-traits (71), while the majority of reports point out that intraindividual variation can depend on environmental and psychological effects (72–74). Another source of variability is the time of the day and time of the year. In an effortful longitudinal study of a single subject over 3.5 years, Choe et al. could show that there were systematic variations with a “significant linear trend, annual periodicity, and persistence” (75). Others have found that resting-state activity varies with the circadian rhythm (76), sleep duration (22), prior events (72), or mood (73). But also the metabolic state of hunger against satiety has a measurable effect on various resting-state measures (77–79)—and the list of factors influencing rs-fMRI and/or the BOLD signal could be continued endlessly.

In summary, while acquisition methods and analysis strategies can be standardized, it will become challenging to control for additional endogenous and exogenous factors in a daily clinical routine. Although all mentioned factors might only have a moderate effect on resting-state measures, in the light of clinical applications, they may be in the same range that differentiates between patients and healthy controls. It is therefore questionable whether rs-fMRI will ever make it into a clinical tool. One might speculate, whether the reliability of task-related fMRI, with concrete tasks that requires focusing the attention, might be more superior and more suitable for clinical application (36, 45, 80).

**Table 1** gives a schematic overview on reliability and current clinical applications of task-related and resting-state fMRI, separated for both single subject and group-level studies.

### Next-Generation Data Analyses

#### Are Meta-Analyses the Solution?

In the light of increasing computational power, cloud computing, and open-access databases with thousands of datasets, meta-analyses became increasingly popular. Meta-analyses are a suitable tool for examining general network structures for a given cognitive task, and which areas, on average, show deviating effects in large patient populations. They may become important cornerstones for building new and more fine-grained models for various disorders.

But, like any emerging new method, the methods behind meta-analyses of large datasets are still under development and standards needs to be established (81). This implies that meta-analyses are not necessarily comparable and may suffer from the publication bias (82). This has been a known issue for decades since it has been noted that meta-analyses and randomized control studies may show different results (83), but methods are under development that control for potential biases (84). Furthermore, pure meta-analyses may not be the most sufficient way to go, since they often provide us only with very general common-sense solutions, that do not go much beyond to those functional lesion maps that already have been drawn in the first half of the last century (85, 86). As a side note, already then, the posterior cingulate cortex has been associated with self-awareness, which is nowadays called the “default mode network.” Or, another example, there is a striking functional and structural similarity between Kleist maps, based on brain lesions and injuries, and the meta-analysis of neuroimaging data on language functions (87).

In summary, meta-analyses are important contributors in revising and updating our understanding of the structural and functional organization of cognitive functions, and how structure and function interact. Focusing either only on lesions or only on fMRI results may not be sufficient enough for building new and more integrative and holistic theories of brain functions and sources of brain disorders. One way of achieving this is, for example, joining structural lesion maps with results from functional imaging within one multivariate analysis (88–90).

#### Is Machine Learning the Solution?

Over the most recent years, meta-analyses have been supplemented with machine- and deep-learning methods that can extract (partly hidden) information out of the data and may be able to detect a pattern that is not observable otherwise. The main characteristic that differentiates deep learning from other classifier approaches, e.g., for identifying subpopulations in a multimodal data space, is that features are learned automatically and do need a feature selection as a preceding step, which removes subjectivity and substantially improves accuracy (91). Deep learning has shown superior performance in detecting cross-modality relations and has attracted a substantial amount of attention among researchers from various fields. Furthermore, it has been nominated as one of the “10 breakthrough technologies” by MIT Technology Reviews (https://tinyurl.com/zx82sg5). Another advantage of deep-learning methods are their depth and breadth in model building, which may uncover hidden relations between factors that are of relevance for future clinical applications of fMRI in psychiatry (92–94).

However, one potential problem with machine-learning approaches might be, however, the problem of overfitting which may compromise generalization of the results (95). Overfitting means that the algorithm finds a solution that perfectly parameterises the given dataset but may fail to classify new data correctly. One reason for overfitting is the use of too-small sample sizes as training data (96). But the field of machine learning has been hit by the replication crisis, as well (97). This is most like caused by insufficiently shared code and (training) data. Accordingly, the use of machine- and deep-learning methods is only justifiable in combination with large-scale open-access databases and open-source software.

In summary, machine-learning approaches are a promising move toward new discoveries of hidden relationships. Once reliable patterns have been identified and validated across different databases, one could expect that these approaches will bring us much closer to clinical applications of single-subject fMRI, as they may allow identifying fingerprints for certain disorders. But, it is still a long way to go until we will see clinical fMRI for diagnostic purposes, since, despite promising progress, the most recent developments are still in their infancies.

## Discussion: the Next Steps in Translational Neuroscience

Translation neuroscience is a rapidly expanding field. If one takes the term literally, it means translating one concept from one domain or scientific discipline into another. In the field of neuroscience, it actually could be thought both ways, either translating a clinical concept into something that is measurable with neuroscientific methods and, the other way around, translating results from neuroscience into clinical praxis. The first way would lead to a better understanding of neurobiological underpinnings of a disorder, while the other way focuses more on the benefits of the patients. However, the issues raised above have to be taken into account for any translational research, whether it is for explorative purpose or diagnostics. The replication crisis might have triggered a new way of think and further attempts to exploring underlying mechanisms. Especially the recent years has seen an increasing interest in exploring all kind of endogenous and exogenous factors that might influence not only brain functions but also the mechanisms that generate the BOLD signal. Some of them have been discussed above, but the list of influencing factors is far from being complete. Moreover, it should highlight that there are indeed several processes that can affect the physiological and vascular basis of the BOLD signal but not necessarily the underlying neuronal mechanisms and activations, like the current blood pressure. Other factors, by contrast, might have a systematic effect on brain functions but do not have anything to do with the neurological or psychiatric disorder that should be examined, like the current phase of the circadian rhythm or the time point of the last meal. Or other factors are purely technical, like temperature, technical noise of the MR system. The influence of these factors might be boosted by the combination with nonstandardized fMRI acquisitions, different instructions (e.g., in rs-fMRI eyes open, eyes closed), nonstandardized analysis methods, less suitable algorithms. While there is an increasing number of reports recommending larger-sample sizes (n > 100) for improving reliability (98), other attempts are needed to improve single-subject fMRI (99), which are compulsory for pushing forward clinical fMRI.

Concerning the aspect of larger sample-sizes, this is mandatory for basic research of neuronal correlates of neurological and psychiatric disorders. Those studies are needed for building models and testing hypothesis of the source of a disorder and its progression. This needs a clear conceptualization of what neurobiological or cognitive components may cause the disorder, and how they can be measured with, for example, neuroimaging. While in some disorders, this might be a rather trivial endeavour, psychiatric disorders are often lacking such a definite relationship. However, machine-learning and big-data approaches may help uncovering hidden relationships and are promising strategies in current research applications (94). An often seen problem within fMRI studies is the huge overlap of results across different cognitive tasks and domains. As pointed out by Hugdahl and coworkers, the fronto-parietal attention network, aka EMN, is virtually activated every time the attention is focused on an extrinsic task (61). Such an unspecific but the fMRI results dominating activation pattern is difficult to interpret in the light of psychiatric disorders where problems may arise in one particular cognitive domain but not in others. Group studies and meta-analyses may provide the sufficient power to study also subtle effects within the EMN that may relate to psychiatric disorders, but the back translation into diagnostic attempts are difficult to achieve. However, one might also have to rethink the concept of certain disorders. Many psychiatric disorders are nowadays formulated and specified as spectrum disorders, forming a gradient from healthy to severely affected with perhaps varying probabilities of certain comorbidities, with unclear aetiologies. Accordingly, it is less likely that one is able to identify one single spot in the brain or one single deviation in a biomarker that causes this particular set of symptoms. Furthermore, there are often unclear and not directly related functional-structural relationships. For example, functional differences seen in rs-fMRI data from patients with depression (100) may not directly correspond to deviations seen in structural data (101). Accordingly, the cognitive and neurobiological models of psychiatric disorders may take into account that the spectrum of a disorder has multiple sources and that structural and functional causes may or may not depend on each other. Unfortunately and despite uncountable neuroimaging studies and meta-analyses, neuroimaging results have yet not been used for redefining and specifying diagnostic categories, as, for example, specified by the DSM5 (4).

Concerning the translation from neuroimaging results in clinical praxis, i.e., using fMRI for diagnostics, this is an even more difficult attempt. As outlined earlier, clinical fMRI is routinely used only for presurgical mapping but not for diagnostic purposes per se, and in particular not for diagnostic purposes in psychiatric disorders. The fMRI signal is too easily affected by many different endogenous and exogenous factors that are difficult to control. Even with standardized acquisition and analysis protocols, substantial and clinically irrelevant variations in individual fMRI results will be still present. Moreover, these variations are most likely at the same magnitude as the deviation from the mean that one would expect in a patient. This problem is further amplified by the fact that many disorders are spectrum disorders with gradual deviations. However, advanced machine-learning approaches that have been applied to large databases achieved, for example, for the classification of autism spectrum disorders already accuracies of 70% (102) to 90% (103). Interestingly, both studies used the same dataset but different algorithms, indicating that the selection of the algorithms can bias the results. Furthermore, there has not been reached a consensus yet, which algorithms are superior or recommended for fMRI data in general or for the classification of specific disorders in particular. But further methodological progress and better standardizations can be expected in the near future.

Besides using fMRI for diagnostics and classification of patients, there have also been attempts at using fMRI for the development of drugs and validating of drug effects (104). However, also this field of translational neuroscience still suffers from the replication crisis, publication bias, and the lack of standard acquisition and analysis methods. Hence, there is currently only very limited applicability of fMRI for this purpose, as well.

## Conclusion

In conclusion, developments and progress have been and will be made in all domains, covered by this article. The replication crisis has pushed the development of new strategies, like the ReproNim initiative, that will help to standardize acquisition and analysis pipelines. Furthermore, the increased computational power and the continually growing number of available open-access databases with large sample-sizes and longitudinal data will allow the generation of “norm”-databases that can describe the distribution and interindividual variability of cognitive functions and network structures. Longitudinal data that are increasingly available will also give a better picture of disease progression. Machine-learning approaches will become better and more reliable in identifying disorders from multiple sources. All these approaches may lead to redefinitions of symptoms and may give a clearer picture of the causes of various spectrum disorders. Whether this will finally lead to clinical fMRI as a diagnostic tool is difficult to predict, since the variability of the BOLD signal is still an unresolved issue. In light of that, rs-fMRI currently does not appear as a tool that shows sufficient reliability and stability within and between subjects. The most reliable way of conducting rs-fMRI might be in combination with a fixation task and at least 12-min scan duration. By contrast, task-related fMRI that require focused attention of the subject might have better reliability and hence predictive value on the single-0subject level but would require a careful selection of clinically relevant paradigms. Here, better theoretical models have to be developed for translating clinical concepts into meaningful fMRI paradigms. Furthermore, it would be beneficial to acquire the data (in particular rs-fMRI) approximately at the same time of the day, and a sleeping and diet protocol could explain further, but irrelevant variability. Meta-analyses, in turn, might help in identifying precisely clinical concepts. In essence, after almost three decades, fMRI has generated substantial new insights into neurological and psychiatric disorders. It has produced a vast amount of data and triggered the development of new methods both for data acquisition and data analysis. Although the reliability of fMRI is still limited and hinders its use for diagnostic purposes in a daily clinical routine, the field of translational neuroscience is continuously moving toward more standardized, more reliable, and more clinical relevant applications of fMRI.

In essence, it is not unlikely to expect that clinical fMRI will at one point go beyond its current presurgical application and toward more diagnostic applications. This will be achieved by improved and standardized methods, better understanding of the neurovascular-coupling mechanisms, and revised models of psychiatric disorders.

## Author Contributions

The author KS did the literature research and wrote the paper without any further assistance.

## Funding

KS is funded through a grant by the Research Council of Norway (276044/ “When default is not default: Solutions to the replication crisis and beyond”).

## Conflict of Interest

The author declares that the research was conducted in the absence of any commercial or financial relationships that could be construed as a potential conflict of interest.

The handling editor declared a past co-authorship with the author KS.
